# Variation in the *CXCR1 *gene (*IL8RA*) is not associated with susceptibility to chronic periodontitis

**DOI:** 10.1186/1477-5751-10-14

**Published:** 2011-11-03

**Authors:** Raquel M Scarel-Caminaga, Karen MC Curtis, Rivelto Renzi, Patrícia M Sogumo, Giovana Anovazzi, Aline C Viana, Yeon J Kim, Silvana RP Orrico, Joni A Cirelli

**Affiliations:** 1 UNESP - São Paulo State University, School of Dentistry at Araraquara, Department of Morphology, SP, Brazil; 2 UNESP - São Paulo State University, School of Dentistry at Araraquara, Department of Oral Diagnosis and Surgery, SP, Brazil

**Keywords:** CXCR1, chemokine, cytokine, genetic polymorphism, periodontal disease

## Abstract

**Background:**

The chemokine receptor 1 CXCR-1 (or IL8R-alpha) is a specific receptor for the interleukin 8 (IL-8), which is chemoattractant for neutrophils and has an important role in the inflammatory response. The polymorphism rs2234671 at position Ex2+860G > C of the *CXCR1 *gene causes a conservative amino acid substitution (S276T). This single nucleotide polymorphism (SNP) seemed to be functional as it was associated with decreased lung cancer risk. Previous studies of our group found association of haplotypes in the *IL8 *and in the *CXCR2 *genes with the multifactorial disease chronic periodontitis. In this study we investigated the polymorphism rs2234671 in 395 Brazilian subjects with and without chronic periodontitis.

**Findings:**

Similar distribution of the allelic and genotypic frequencies were observed between the groups (p > 0.05).

**Conclusions:**

The polymorphism rs2234671 in the *CXCR1 *gene was not associated with the susceptibility to chronic periodontitis in the studied Brazilian population.

## Findings

The human chemokine receptor CXCR-1 (or IL8R-alpha) is a specific receptor for the chemokine interleukin 8 (IL-8) [1]. Initially identified as a chemoattractant for neutrophils, IL-8 has been demonstrated to have pro-inflammatory effects including stimulation of neutrophil degranulation [2]. Cellular activities of IL-8 are mediated by CXCR-1 and CXCR-2 (IL8R-beta), which maintain 78% of amino acid similarity and are encoded by two single-copy genes that are located on chromosome 2q34-35 [3]. However, CXCR-1 is more specific for IL-8 in comparison with CXCR-2 [4].

The involvement of IL-8, CXCR-1 and CXCR-2 has been extensively investigated in different diseases such as pyelonephritis [5,6], hepatitis B [7], rapid disease progression of HIV-1^+ ^[8], lung diseases, such as chronic obstructive pulmonary disease and asthma [9], bronchiectasis [10], systemic sclerosis [11] and lung cancer [12]. Some of these studies have reported positive associations between the diseases and single nucleotide polymorphisms (SNPs) in the *CXCR1 *gene [8,9]. Indeed, a significant association was demonstrated between the 860G > C (S276T) SNP in the *CXCR1 *gene with decreased lung cancer risk [12]. Haplotypes formed by SNPs in the *CXCR1 *and *CXCR2 *genes where also previously identified [7].

The 860G > C (S276T) SNP in *CXCR1 *gene was identified by comparison of multiple sequences deposited in the GenBank/EMBL data banks [11,13]. These authors named this polymorphism differently: +2607 (position 6334 of sequence accession number [GenBank: L19592.1]) in exon 2, and +827 (starting from the initiation of the ATG codon in exon 2 of [GenBank: L19592.1]), respectively. The variant position 860G > C is based on the [NCBI: NM000634.2] exon 2 initiations, however it is important to be clear that all these different positions at the *CXCR1 *gene refer to the same polymorphism (G > C), which results in a conservative amino acid substitution from serine to threonine at the 276 amino acid residue of the CXCR-1 (or IL8R-alpha) protein. Here, we preferred to use the reference sequence number [refSNP ID: rs2234671] in NCBI's Entrez system (http://www.ncbi.nlm.nih.gov/SNP).

Predominantly in the last decade, many candidate-gene investigations have been conducted in order to find genetic risk factors associated with chronic periodontitis (CP). Epidemiological studies indicate that CP is an important cause of teeth loss in adults, since 5% to 15% of any population suffers from this disease [14]. CP is a multifactorial disease, initiated by bacterial infection which can progress to the damage and destruction of the supporting tissues of the teeth [15]. The host response is influenced by both environmental (e.g. smoking, oral hygiene) and genetic factors [16]. Some studies have demonstrated association between polymorphisms in genes of the immune system and CP, such as *Interleukin 2 *(*IL2*) [17,18], *IL4 *[19-21], *IL6 *[22,23] and *IL10 *[24,25]. Recently, we have reported association between haplotypes in the *IL8 *and in the *CXCR2 *genes with CP [26,27]. Because those previous findings and the biological relationship of the CXCR-1 with the IL-8 and the CXCR-2 [1,5] we have hypothesized whether a SNP in the *CXCR1 *gene would also influence the host susceptibility to CP.

In this regard, the aim of this study was to investigate the association of the rs2234671 SNP in the *CXCR1 *gene in a Brazilian population with chronic periodontitis.

## Materials and methods

In total, 395 individuals in good general health and having similar socioeconomic status were recruited from the patient pool of the School of Dentistry at Araraquara, São Paulo State University - UNESP between November 2004 and May 2007. The study was approved by the Committee for Ethical Affairs of the São Paulo State University (Protocol number 57/04).

To verify statistical power of our sample, we used the G*POWER 3 software [28] with the following parameters: logistic regression; two tail, odds ratio = 1.56, α error probability = 0.05. Detailed clinical criteria for include patients in this study are described in Viana et al. [27]. Briefly, the following clinical signs and parameters were assessed at six sites around each tooth: probing pocket depth (PPD) (measured as distance from the free gingival margin to the base of the pocket), clinical attachment loss (CAL) (as the distance from the cement-enamel junction to the base of the periodontal pocket) and bleeding on probing (BOP) (registered as present or absent). These measurements were performed in millimeters using a periodontal probe with Williams markings. All fully erupted teeth, except third molars and retained roots, were examined. The diagnosis of subjects was established on the basis of clinical criteria proposed by the 1999 International World Workshop for a Classification of Periodontal Diseases and Conditions [29]. The subjects were categorized into two groups: controls (n = 191), subjects exhibiting no sites with CAL and PPD ≥ 3 mm and no BOP; and CP (n = 200), subjects exhibiting one or more sites with CAL and PPD ≥ 3 mm and BOP.

Information on smoking status was obtained using a self-reported questionnaire, and subjects were classified as a "smoker" or "nonsmoker" according to Kornman et al.[30].

DNA was obtained from buccal epithelial cells, extracted with sequential phenolchloroform/isoamyl alcohol (25:24:1) solution and precipitated with a salt ethanol solution. The SNP was identified using the sequence-specific primer-polymerase chain reaction method (SSP-PCR) according to Renzoni et al. [11]. The PCR products were electrophoresed (100 V, 120 min) in 10% polyacrylamide gels and visualized by silver staining.

The chi-squared test was used to determine whether the groups were composed of patients with the same proportion of males and females and whether they had similar smoking habits. Onset age distribution between the two groups was evaluated by Students't-test. Those statistical analyses, as well as deviations from Hardy-Weinberg equilibrium, were performed using BioEstat software version 5.0 (UFPA, MCT, CNPq, Belém, PA, Brazil).

In addition, a forward stepwise multiple logistic regression analysis was used for estimating the relationships between the SNP rs2234671 in the *CXCR1*gene and periodontal disease susceptibility and the other covariates. This multivariate logistic regression modeling was executed using the R statistical package (R Development Core Team, Vienna, Austria). Differences were considered to be significant when p < 0.05.

## Results

The power calculations showed that the sample size of 395 individuals demonstrated a power of 93%. Therefore, the number of subjects enrolled in this study is large enough to detect association with an acceptable level of confidence. The population investigated here was composed mainly of female subjects (59.7%) and nonsmokers (84.8%) and the mean age of the individuals was 39.49 years (Table 1). The minor allele frequency (MAF) was 0.087, and the genotype distribution of the rs2234671 SNP in the *CXCR1 *gene was consistent with the assumption of the Hardy-Weinberg equilibrium for the control (p = 0.1719) and CP groups (*p *= 0.2777).

**Table 1:** Characteristics of the studied populations

	Control n = 195	Periodontitis n = 200	Total n = 395	p
**Age**, mean (±)	35.47(± 9.9)	43.44(± 10.5)	39.49(± 10.9)	< 0.0001^a^
**Gender **n (%)				
Female	114 (58%)	122 (61%)	236 (59.7%)	0.74^b^
Male	81 (42%)	78 (39%)	159 (40.3%)	
**Smoking habits **n (%)				
Nonsmokers	179 (91%)	156 (78%)	335 (84.8%)	< 0.0002^b^
Smokers	16 (9%)	44 (22%)	60 (15.2%)	

^a ^Student t-test; ^b^Chi-squared test

Significant associations between age and periodontitis were observed by univariate analysis, mainly considering the ORs for age groups 30-39 to the 50-59. We consider that besides the 60-69 and > 70 age groups showed significant OR values they were uncommon and demonstrated a wide range of CI. Significant association was also found between smoking status and periodontitis (OR = 3.8). Therefore, age and smoking status were considered confounding factors. Even though gender was not found to be associated with periodontitis, we included it in the confounding factors in the multivariate analysis, to adjust for any small confounding effects. To more accurately evaluate the strength of any association and to eliminate the distortion caused by confounding effects, multivariate analysis was performed. The multiple logistic regression analysis demonstrated that neither genotypes nor alleles were associated with periodontitis, even after adjusting for covariates, including age, gender and smoking status (Table 2). Therefore, this polymorphism could not be considered a genetic risk for CP in the studied Brazilian population.

**Table 2:** Regression logistic results of the analysis

Characteristics of patients		Control n = 195 (%)	Periodontitis n = 200 (%)	P-Value	OR (95% CI)
Age	20-29	72(79.78)	19(20.22)		Reference
	30-39	59(51.75)	55(48.25)	7.73e-05	3.47 (1.82-6.63)
	40-49	48(37.21)	81(62.79)	5.89e-10	6.42 (3.4-12.1)
	50-59	13(30.96)	29(69.04)	1.14e-07	8.46 (3.64-19.63)
	60-69	2(14.29)	12(85.71)	4.94e-07	23.3 (5.19-104.65)
	> 70	1(20)	4(80)	3.67e-03	13.77 (1.67-113.32)
					
Gender	Male	81(50.94)	78 (49.06)		Reference
	Female	114 (48.31)	122 (51.69)	0.65	1.11 (0.7-1.74)
					
Smoking habits	Nonsmokers	179 (53.42)	156 (46.58)		Reference
	Smokers	16 (26.67)	44 (73.33)	2.203134e-05	3.8 (1.97-7.33)
					
Polymorphism rs2234671	GG	164 (84.1)	166 (83.3)		Reference
	CG	28 (14.4)	31(15.5)	0.55	1.2 (0.65-2.2)
	CC	3 (1.5)	3 (1.5)	0.89	1.13 (0.18-7.27)
					
	G	356 (91.3)	363 (90.8)		Reference
	C	34 (8.7)	37 (9.2)	0.52	1.21 (0.68-2.15)

## Discussion

To our knowledge, this is the first time that the rs2234671 SNP in the *CXCR1 *gene was investigated regarding the CP. Single nucleotide polymorphisms are mainly useful in studies of human population genetics and candidate-gene studies for disease association [31]. This makes possible the development of the susceptibility profile concept for specific diseases, like the risk of Alzheimer's, which has been considered to be substantially influenced by a total of ten genetic polymorphisms of inflammation-related molecules [32]. If the high-susceptibility profile for CP be determined, genetically susceptible subjects would be identified earlier and therapeutic intervention strategies could be envisaged aiming prevention of disease establishment. However, many studies with different populations would be needed to reach a high-risk profile for periodontal disease[16].

Another important point to be considered in studying genetic variations is about the functionality of a polymorphism. Considering that the rs2234671 SNP causes a conservative amino acid substitution (S276T) in the third extracellular loop of the CXCR-1 protein, one can suppose whether this event would influence the ligand/binding interaction of the chemokine receptor. This speculation was raised by Liu and colleagues [33], which found four SNPs (including the rs2234671) in human populations, that were not found in nonhuman primate species when the gene sequences were compared. The authors concluded that there is an accelerated CXCR-1 protein evolution in the human lineage. However, more studies are necessary in order to clarify whether this SNP is functional, and whether it would be under selective pressure aiming to improve innate immunity.

Relevant factors known to influence the pathogenesis of periodontitis were assessed by multivariate analysis. Regarding age, there is evidence that both the prevalence and severity of periodontitis increase with increasing age [34]. This can be explained by the cumulative effect of prolonged exposure to other risk factors [35]. There is a multitude of studies (more than 325) that have shown a relationship between smoking and periodontitis [36]. In the present study, smoking was found as a risk factor for periodontitis (OR = 3.8, 95% CI: 1.97-7.33). Interestingly, Tonetti et al.[37] reported that cigarette smoking is associated with two- to threefold increases in the odds of developing periodontitis. Also similar with our results, Tomar & Asma [38] found that current smokers were about four times as likely to have periodontitis than persons who never smoked, after adjusting for covariates such as age, gender and education. Some studies have shown that smokers had significantly worse clinical symptoms of periodontitis than non-smokers [39,40].

We did not take into account ancestry in our study because we only obtained the skin color information. Although Brazilian population is admixtured, the skin color is not related with ancestry according to Pena et al.[41], Parra et al.[42] and Pimenta et al.[43].

In regards to our hypothesis whether the rs2234671 SNP in the *CXCR1 *gene would be associated with susceptibility to chronic periodontitis, the obtained results showing a lack of association permit us to conclude that this SNP was not useful as a genetic risk factor for CP in the studied Brazilian population.

## Abbreviations

IL-8: Interleukin 8; SNP: Single Nucleotide Polymorphism; CP: Chronic Periodontitis; PPD: Probing Pocket Depth; CAL: Clinical Attachment Loss; BOP: Bleeding on Probing.

## Competing interests

The authors declare that they have no competing interests.

## Authors' contributions

RMSC conceived and coordinated the study, performed statistical analysis and wrote the manuscript. KMCC, RR, PMS: carried out the molecular reactions and helped analyze the results. GA: helped with the statistical analysis and helped wrote the manuscript. ACV, YJK: examined and selected the patients and extracted the DNA. SRPO, JAC: participated in the study design, defined the clinical criteria of the studied groups and helped wrote the manuscript. All authors read and approved the final manuscript.

## Acknowledgements

This study was supported by CAPES and FAPESP grants (03/10424-0, 06/04492-1, 05/03231-7, 2005/04553-8).
